# Emergency ventilator for COVID-19

**DOI:** 10.1371/journal.pone.0244963

**Published:** 2020-12-30

**Authors:** William P. King, Jennifer Amos, Magdi Azer, Daniel Baker, Rashid Bashir, Catherine Best, Eliot Bethke, Stephen A. Boppart, Elisabeth Bralts, Ryan M. Corey, Rachael Dietkus, Gary Durack, Stefan Elbel, Greg Elliott, Jake Fava, Nigel Goldenfeld, Molly H. Goldstein, Courtney Hayes, Nicole Herndon, Shandra Jamison, Blake Johnson, Harley Johnson, Mark Johnson, John Kolaczynski, Tonghun Lee, Sergei Maslov, Davis J. McGregor, Derek Milner, Ralf Moller, Jonathan Mosley, Andy Musser, Max Newberger, David Null, Lucas O’Bryan, Michael Oelze, Jerry O’Leary, Alex Pagano, Michael Philpott, Brian Pianfetti, Alex Pille, Luca Pizzuto, Brian Ricconi, Marcello Rubessa, Sam Rylowicz, Clifford Shipley, Andrew C. Singer, Brian Stewart, Rachel Switzky, Sameh Tawfick, Matthew Wheeler, Karen White, Evan M. Widloski, Eric Wood, Charles Wood, Abigail R. Wooldridge

**Affiliations:** 1 Grainger College of Engineering, University of Illinois Urbana-Champaign, Urbana, IL, United States of America; 2 Carle Illinois College of Medicine, University of Illinois Urbana-Champaign, Urbana, IL, United States of America; 3 Applied Research Institute, University of Illinois Urbana-Champaign, Urbana, IL, United States of America; 4 Fast Radius, Chicago, IL, United States of America; 5 Siebel Center for Design, University of Illinois Urbana-Champaign, Urbana, IL, United States of America; 6 Tekmill, Champaign, IL, United States of America; 7 Creative Thermal Solutions, Urbana, IL, United States of America; 8 College of Veterinary Medicine, University of Illinois Urbana-Champaign, Urbana, IL, United States of America; 9 Carle Foundation Hospital, Urbana, IL, United States of America; 10 College of Agricultural, Consumer, and Animal Sciences, University of Illinois Urbana-Champaign, Urbana, IL, United States of America; University of Alberta, CANADA

## Abstract

The COVID-19 pandemic disrupted the world in 2020 by spreading at unprecedented rates and causing tens of thousands of fatalities within a few months. The number of deaths dramatically increased in regions where the number of patients in need of hospital care exceeded the availability of care. Many COVID-19 patients experience Acute Respiratory Distress Syndrome (ARDS), a condition that can be treated with mechanical ventilation. In response to the need for mechanical ventilators, designed and tested an emergency ventilator (EV) that can control a patient’s peak inspiratory pressure (PIP) and breathing rate, while keeping a positive end expiratory pressure (PEEP). This article describes the rapid design, prototyping, and testing of the EV. The development process was enabled by rapid design iterations using additive manufacturing (AM). In the initial design phase, iterations between design, AM, and testing enabled a working prototype within one week. The designs of the 16 different components of the ventilator were locked by additively manufacturing and testing a total of 283 parts having parametrically varied dimensions. In the second stage, AM was used to produce 75 functional prototypes to support engineering evaluation and animal testing. The devices were tested over more than two million cycles. We also developed an electronic monitoring system and with automatic alarm to provide for safe operation, along with training materials and user guides. The final designs are available online under a free license. The designs have been transferred to more than 70 organizations in 15 countries. This project demonstrates the potential for ultra-fast product design, engineering, and testing of medical devices needed for COVID-19 emergency response.

## Introduction and background

The infectious disease COVID-19, caused by a novel coronavirus, began in China in December 2019 [1] and in the subsequent months the disease swept rapidly across the world. By the time that the World Health Organization announced that the disease had become a pandemic on March 11, 2020 [2, 3], there had been 118,000 cases of the disease reported in 114 countries. A key concern about the disease is the potential for exponential growth of the number of patients and the possibility that heath care infrastructure could be overwhelmed [4–6]. COVID-19 patients can experience acute respiratory distress syndrome (ARDS), a condition where a patient has extreme difficulty breathing due to fluid leaking into the lungs [7–9]. A patient with ARDS can be treated with mechanical ventilation, the goal of which is to provide oxygen to the patient while the underlying disease runs its course [7, 10]. Appropriate oxygen delivery is a mainstay of critical care, and in COVID-19, death from ARDS and hypoxemia is treated in the critical stages with mechanical ventilation. When a patient requires a ventilator and one is not available, the patient’s life could be at risk. While some publications report that mechanical ventilation is not correlated with high rates of survival in COVID-19 patients [7–9, 11], ventilation of COVID-19 patients is recommended by the Society of Critical Care Medicine [7] and the American Association for Respiratory Care (AARC) [12].

There are many mechanical ventilators on the market with different levels of complexity and sophistication [13, 14]. The most sophisticated hospital ventilators have integrated sensors, electronics, and software intelligence that control the volume of air flow, air pressure, and breathing rate. Because of the exponential growth of COVID-19 infected patients, it is currently unknown whether enough hospital-grade ventilators will be available to meet demand in the coming weeks and months [6, 15, 16]. When a hospital-grade ventilator is not available, alternative ventilation methods are desired.

A *low function ventilator* is an alternative to a hospital grade ventilator that is simpler, less expensive, and has lower capability for controlling air flow during ventilation [12, 17–21]. There are a variety of low function ventilators that are commercially available, with different ventilators designed for different applications and intended to satisfy the requirements of those applications [19]. An *automatic resuscitator* is a device that can replace hand-bagging to provide oxygen to a patient who has stopped breathing, a *portable ventilator* or *transport ventilator* is capable of ventilation outside of a hospital or while the patient is being moved, and a *disaster ventilator* or *emergency ventilator* is a device that is deployed in an emergency when there are patients that need a ventilator but a hospital grade ventilator is not available [18, 19, 22–26]. While low function ventilators all have lower capability than a conventional hospital grade ventilator, they can be attractive for certain situations where the attributes of low cost, simplicity, and accessibility are important. We refer to the device of this study as an emergency ventilator or EV, because of its intended use in an emergency and because we demonstrate ventilation of an animal using the device.

The COVID-19 emergency has highlighted the need for research into EVs [27–29]. In general, there is a lack of peer-reviewed articles that describe the science and engineering of EV design and performance, and there is a need for new research into EV design, performance, and testing. This paper reports design, functional testing, integrated electronic monitoring, and animal testing of a gas-powered pressure-switched EV. We also describe a methodology by which a large interdisciplinary team worked together in an emergency.

### Design and engineering

The project began with discussions between engineers, physicians, scientists, and designers to define a problem statement. There was some uncertainty about the number of ventilators that might be needed in the local community, state, and nation. Our team included experts on computational biophysics who provided projections for the number of hospital patients that could be expected under different conditions such as rate of spread in our community and beyond [5]. The methodology was to solve the differential equations describing a Susceptible-Exposed-Infected-Recovered (SEIR) model, calibrated to hospital data in the early stages of the pandemic, using a customized code [30]. The calculations solve the differential equations of the SEIR model without spatial extension, demographic stochasticity, or attention to small-world and scale-free network effects, but these are potentially important [31, 32] and could be readily added in the future [33–37]. The model additionally has compartments for severely sick people who are hospitalized, people in critical condition in need of intensive care rooms and ventilators, and a fatal category. The simulation uses severity assumptions as a function of individual age, informed by epidemiological and clinical observations in China [38]; no modifications were made for national differences, such as number of smokers in the population and other nation-specific factors. The model was calibrated using hospital data in the Chicago, IL area and was able to account for the rapid rise in COVID-19 patients during the first two weeks of March. Similar methodologies have been used by other researchers to model the early stages of the COVID-19 epidemic [4]. In addition to the modeling, we consulted with local and regional health care providers about the number of ventilators available. The team decided that in order to prepare local communities for a worst-case scenario, we needed the ability to obtain 1,000 ventilators within 3 weeks and 8,000 ventilators within about 6 weeks if possible. We also understood that there were ventilators needed in other communities and other parts of the world which could require many more ventilators.

By March 16, 2020, there were no ventilators available for commercial sale from medical supply distributors. Most distributors could not provide guidance on when ventilators would be available for purchase. In the few cases where guidance was provided, the team was told that ventilators would be available in six to twelve weeks. We contacted ventilator manufacturers, who were unresponsive to repeated requests to purchase devices and for general requests for help. The ventilator manufacturers were understandably overwhelmed by a surge in demand. Finding no other solution, the team chose to design an EV. An EV was selected over a more sophisticated ventilator because the extreme simplicity of an EV would allow for rapid development and the potential to quickly scale up manufacturing. From this point on, the product was referred to as the Illinois RapidVent, due to the speed at which the project was moving as well as the speed at which a simple EV can be deployed for patient care.

**Fig 1:**
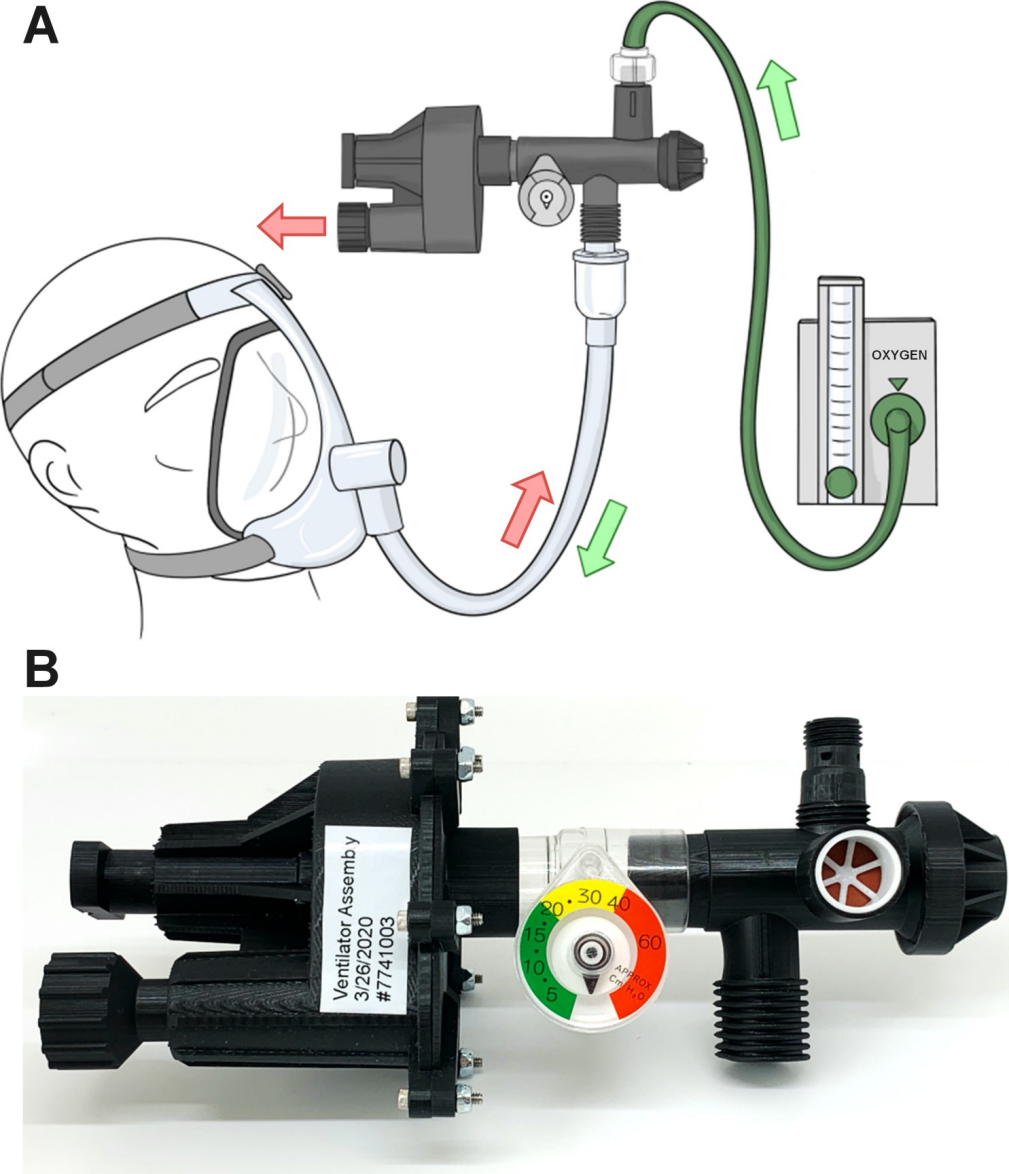
(A) Schematic of the RapidVent connected to oxygen source (green arrow) and to the patient. A single tube carries the inhaled and exhaled air (pink arrow). (B) Photograph of the RapidVent prototype that was used for the various tests in this study. The middle transparent section and the manometer dial are off the shelf parts.

To understand how an EV would be used, we interviewed critical care physicians and respiratory therapists at Carle Hospital in Urbana, IL and developed the functional requirements as follows. The EV must: (1) control the Peak Inspiratory Pressure (PIP) over the range of 20–40 cm H_2_O; (2) operate with a Positive Expiratory End Pressure (PEEP) up to 12 cm H_2_O; (3) operate with maximum volumetric flow rate of at least 15 L/min; (4) operate with breathing rate of 10–30 breaths per minute (BPM); and (5) operate for at least 24 hours continuously and much longer if possible. The PIP and PEEP requirements were particularly important, as patients suffering from ARDS can require higher than normal pressure [7–11]. Hospital personnel also requested that the EV be able to interface with the existing hospital infrastructure. The hospital rooms have 50 psi oxygen supply mounted on the wall, with an integrated flowmeter and regulator. The EV could therefore be used to upgrade the regular hospital rooms beyond their current level of capability or alternatively be used with a portable oxygen tank in an emergency field hospital. Physicians requested that the EV interface with a heat and moisture exchange (HME) filter to control the humidity of the air the patient would breathe, and a HEPA filter to capture aerosolized virus particles coming from the patient. Physicians also requested the EV be able to interface with a standard endotracheal tube as well as a bilevel positive airway pressure (BiPAP) mask. The filters, tubes, and masks are standard equipment available in many hospitals in the United States. Fig 1 shows the RapidVent connected to the oxygen supply available in a hospital room and connected to a patient through an endotracheal tube or mask.

**Fig 2:**
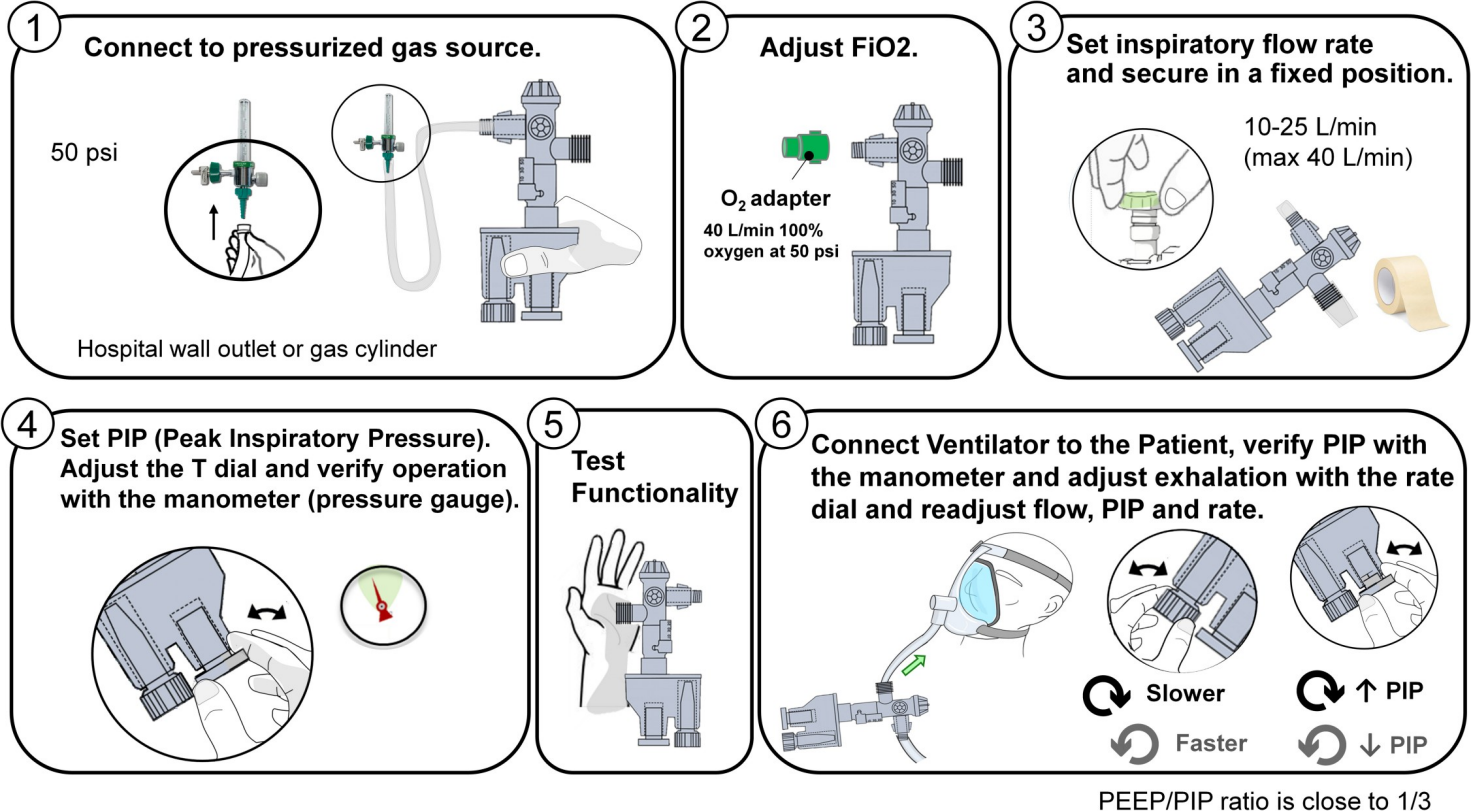
Training materials showing the steps of using the RapidVent.

Physicians and respiratory therapists also described how they would use the EV. The location, size, and labeling of the control knobs regulating oxygen flow are particularly important. The ventilator should be oriented in a way that the device functions as intended while also allowing a caregiver to easily control the device. Appropriate training would maximize the effective and safe deployment of the devices to patients in need, both in the regular hospital setting as well as a potential field hospital setting. We developed documents and videos that could be used to train caregivers. Fig 2 is an example of one of the user aids that was developed.

### Working principles and functional design

The RapidVent is powered by gas pressure from the oxygen source and uses a valve to cycle between inhalation and exhalation at specific pressures set by the operator. During inhalation, oxygen flows through the ventilator and into the patient’s lungs, while the internal pressure increases until the maximum pressure at the end of inspiration. This maximum pressure is the Peak Inspiratory Pressure (PIP). At the PIP, a value opens to allow expiration. The patient’s lungs contract to push air from the device and the internal pressure decreases until the minimum pressure at the end of the exhalation period. The minimum pressure is the Positive Expiratory End Pressure (PEEP). At the PEEP, the valve closes and the cycle repeats. During exhalation, the oxygen supply continues to flow through the device and out through the exhalation port, which evacuates any CO_2_ from the device. The user can set the PIP through a dial that modulates the preloading force on the valve. The user can also set the expiratory time through a dial that sets the rate of exhalation. Lower exhalation rate results in fewer breaths per minute; higher exhalation rate results in more breaths per minute. The PEEP is intrinsic to the design of the device as a fixed ratio with the PIP. There are two safety mechanisms designed into the RapidVent. The first is a one-way value in the body of the RapidVent that opens to room air when the patient attempts to inhale on their own. The second is a valve that opens if the internal pressure exceeds a set value; this pressure threshold set through the mechanical design of the device and in our case was designed to be 45 cm H_2_O.

In order to compare the RapidVent with other EVs, we found and purchased three EVs from secondhand online sources [20, 21]. These devices were available for purchase because they had expired and were unusable for patient care. The EVs were inspected carefully, and in some cases the dimensions of the components were measured with calipers and optical scanners. Key dimensions were extracted using metrology software [39]. The EVs were also tested for comparison with the RapidVent, described later.

**Fig 3:**
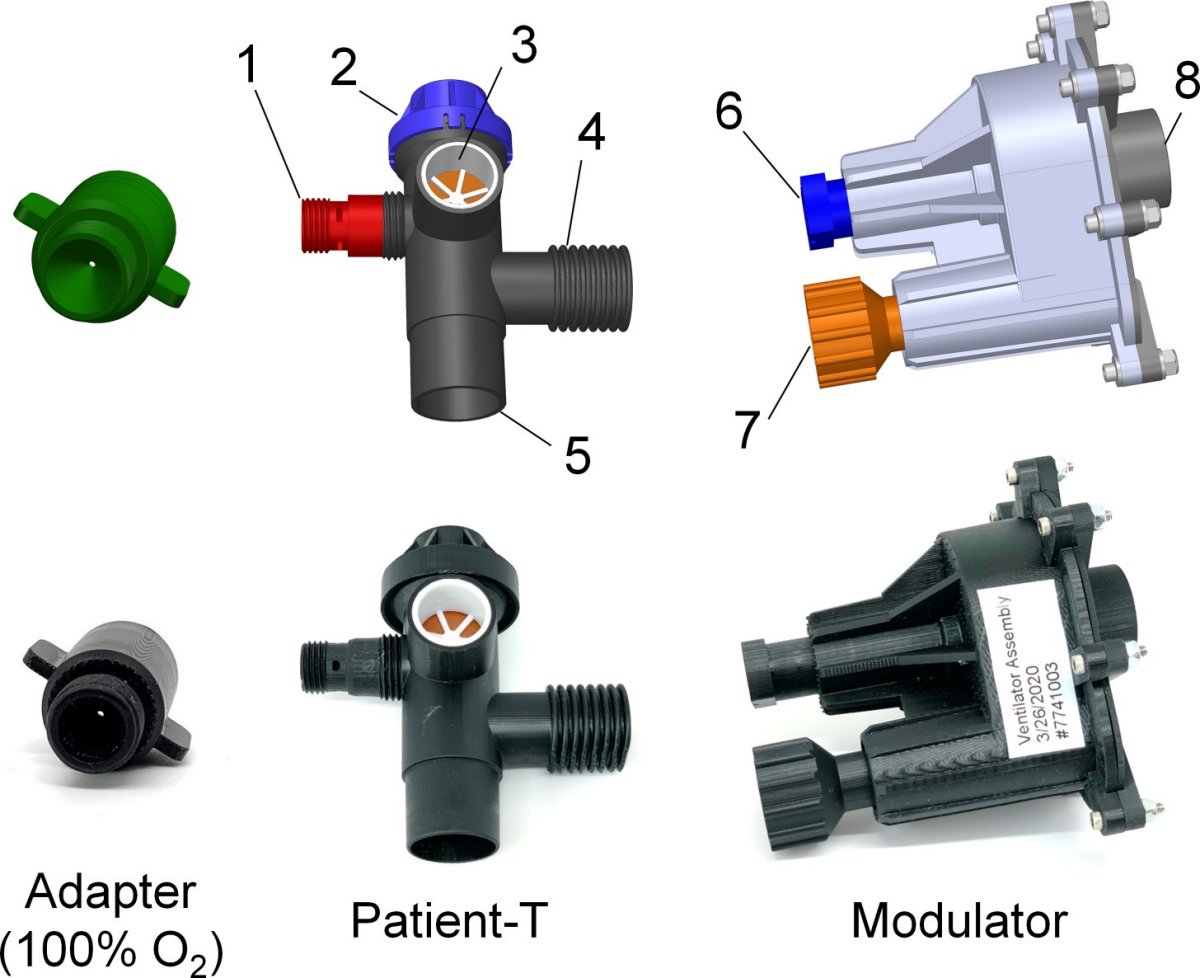
**CAD (top) and photographs (bottom) of the various parts of the RapidVent prototype**. (1) Connection to O2 and FiO2 air entrainment nozzle; (2) pop-off pressure relief valve; (3) one-way valve; (4) connection to patient; (5) connection to the rest of the ventilator; (6) Peak Inspiratory Pressure (PIP) dial; and (7) rate dial.

The RapidVent has three sub-systems: the patient-T, the manometer section, and the modulator. Fig 3 shows two of these sections and the supporting information shows the Bill of Materials (S1 Fig) as well as an exploded view of the CAD (S2 Fig). The sub-system terminology is based on that of a commercial device was used to compare the performance of the RapidVent [40], The patient-T allows the patient to be connected to the RapidVent using a 22 mm barb fitting or 15 mm diameter internal taper, which is standard for ventilation tubing. This dimensional standard also allows the caregiver to attach additional filters, sensors, or other devices to the ventilation tube. The ventilation tubes between the RapidVent to the patient as short as possible, because long tubes can reduce patient ventilation due to dead space within the tubing. There are two options for attaching the supply oxygen to the RapidVent. The first is an entrainment nozzle that mixes oxygen with air pulled from the room. The second option is a cap that delivers 100% oxygen. In either case, the gas flow rate is as high as 40 L/min when attached to a 50 psi gas supply. A narrow orifice restricts the gas flow and drops the pressure from the 50 psi inlet to a near atmospheric pressure inside the ventilator.

The second section of the RapidVent is the manometer section that links together the two major sections of the device and provides for a connection to a sensor that indicates whether the patient is breathing. An analog manometer is installed in the RapidVent with pressure level readings between 0 and 60 cm H_2_O, the effective pressure range for the device. The sensor need not be highly accurate, as its main purpose is to indicate whether the patient is breathing, and to provide an approximate value of PIP and PEEP during pressure cycling. The team evaluated both dial readout and spring calibrated pressure gauge alternatives for the manometer. The manometer section has a medical standard 22 mm male and female tapered fitting on each end.

**Fig 4:**
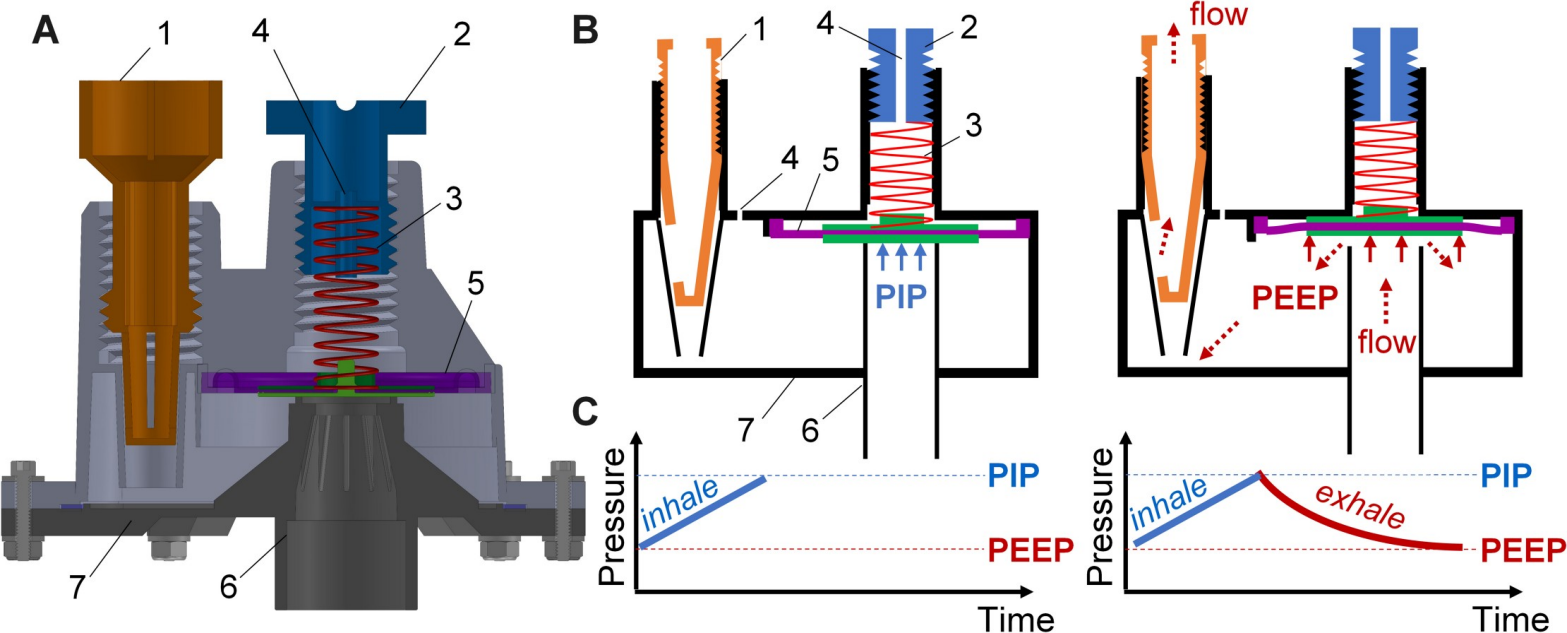
Principle of operation of the RapidVent. (A) Cross section of the modulator design. (1) The breathing rate dial and exhaust port. (2) The Peak Inspiratory Pressure (PIP) dial. (3) Linear spring. (4) Passive pressure relief holes. (5) The diaphragm. (6) The modulator tube. (7) The modulator enclosure. (B) Schematic showing the mechanism of pressure-driven ventilation during inhalation (left) and exhalation (right). During inhalation, the modulator tube is sealed by the diaphragm. After the PIP is reached, the diaphragm moves up allowing exhalation to start. The lung pressure is released until the Positive End Expiratory Pressure (PEEP) value is reached. At the PEEP point, the diaphragm moves back down and seals the tube. The cycle repeats. (C) Pressure versus time during the inhalation (left) and exhalation (right) half cycles.

Fig 4 shows the modulator which provides pneumatic control. The tube from the manometer extends into a chamber where the end of the tube is sealed by a diaphragm, held in place by a spring. The diaphragm is much larger than the diameter of the tube and has a compliant circumference (silicone) and hard plastic center for sealing against the tube and minimizing wear of the spring. The compliant portion of the diaphragm has a geometry that allows the disk to be centered and sealed against the tube. As pressure builds during inspiration, the pressure in the tube creates a force on the diaphragm. When the force on the diaphragm exceeds the force of the compressed PIP spring, the diaphragm moves to unseal the tube and device switches. The rate dial acts as a needle valve, setting how quickly the chamber exhausts the gas, therefore controlling the expiration time in the breathing cycle. The PEEP is intrinsic to the design, set by the ratio of the diaphragm area to the tube cross-sectional area, the diaphragm compliance, and the spring constant. As the pressure decreases, the force on the diaphragm also decreases, and the PIP spring forces the diaphragm into the original position, resetting the cycle.

### Additive manufacturing

Additive manufacturing was used for rapid development and to make prototypes. In the first phase focusing on development, iterations were made on the design to achieve the required functionality of each sub-system as fast as possible until the final design was locked. In the second phase, 75 fully functional prototypes were produced for testing. Manufacturing equipment from Carbon (Carbon M2, Redwood City, CA) was used to print the parts, because of the high production rates possible on these machines, as well as the ability to produce engineering grade materials that would result in high quality parts and an engineering evaluation of EV function [41].

During the development phase, the engineering team evaluated multiple designs in a parallel, rather than sequential process. For instance, several models for each part were designed by parametrically varying critical dimensions and were printed in the same build. This approach proved particularly useful for optimizing multiple parts, especially the diaphragm which is critical for pressure switching the EV. The team designed and printed 40 different diaphragm designs in 8 build on 8 Carbon machines working in parallel. The diaphragm material is Sil-30, which provides the softest available material on the Carbon machine. The various designs explored varying the thickness of the membrane, its geometry, and its external diameter which provides lateral sealing in the modulator body. The diaphragms were tested until the design that gave the best performance was identified and used later in the next phase of the project.

The roughness and dimensional accuracy of AM parts is critical for applications requiring sealing. We were able to obtain extremely smooth surfaces required for adequate seals by carefully orienting the parts during the build. For instance, surfaces in contact with the build platform or support surfaces have higher roughness and some dimensional variability, and this leads to poor sealing. To alleviate this problem, surfaces requiring low roughness were printed such that they are facing away from the build plate, i.e. they are free surfaces during printing and do not contact any other surface. This approach gave very good sealing for the silicone in the one-way valve and the flat surface of the plunger used to seal the pop-off valve. The good performance came at the cost of using excessive support materials to orient the parts to get the critical surfaces to be free. Both parts were printed using UMA as it provides a suitable modulus for sealing applications. In addition to the silicone components, other critical components requiring multiple tests included the threaded connections on the patient-T and modulator as well as interlocking tapered cylinders in the manometer region.

Overall, the total number of parts during the initial development phase was 283, which took 66 hours to print, divided over 43 builds. Once the design was locked, 75 fully functional prototypes were produced during the production phase. The total number of AM parts in each prototype is 15. The 75 prototypes were produced in a total number of 247 builds which lasted a total duration of 400 hours.

### Functional testing

**Fig 5:**
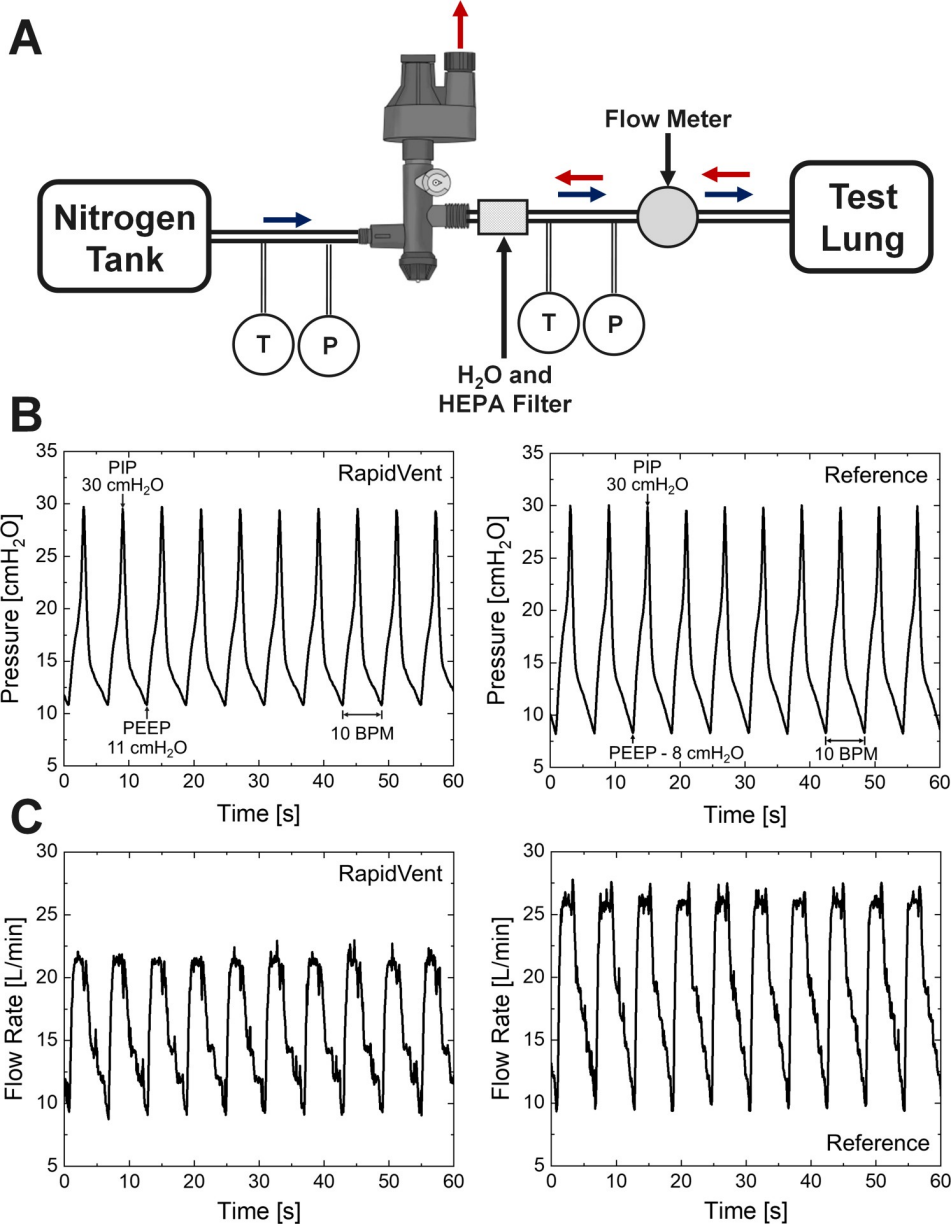
The testing setup and measured performance of the RapidVent. (A) Schematic of the testing setup. “T” and “P” refer to temperature and pressure sensors. (B) Pressure measured between the ventilator and the test lung versus time on the RapidVent (left) and a commercial EV for reference (right). (C) Flow rate measured between the ventilator and the test lung versus time on the RapidVent (left) and the reference device (right). BPM is Breaths Per Minute.

The performance of the prototype EVs and their suitability for clinic scenarios was evaluated with benchtop functional testing. The devices were connected to a 50 psi air source from an oil-free compressor, which was used instead of oxygen to initially test the devices. Pressure gauges (Emerson Rosemount 1511) and thermocouples (Omega Type T) measured the gas at both the inlet and exit of the EV, as shown in Fig 5. A flow rate sensor (Sierra 730) between the ventilator and the patient lung monitored instantaneous flow rates into and out of a test lung. Tidal volume can be calculated from these measurements.

Fig 5 shows the performance of the RapidVent as well as the performance of a commercially available EV tested for comparison purposes. The PIP was set to 30 cm-H_2_O and respiratory rate was set 10 BPM (breaths per minute). The devices have similar performance except for one important difference. The PEEP of the RapidVent is 11 cm-H_2_O versus 9 cm-H_2_O for the reference design. The PEEP to PIP ratio of the RapidVent was tailored to be approximately 1/3, somewhat less than some commercial EVs that have a ratio of 1/5. The RapidVent was designed for higher PEEP levels than commercial EVs, as COVID-19 patients can require PEEP levels in the range 10–15 cm-H_2_O and PIP levels in the range 30–40 cm-H_2_O [9]. The flow rate of the RapidVent is 0–22 L/min versus 10–27 L/min for the reference design. The difference is flow rate is in part due to the differences in PEEP, which affects the air volume required to come to the desired PIP.

**Fig 6:**
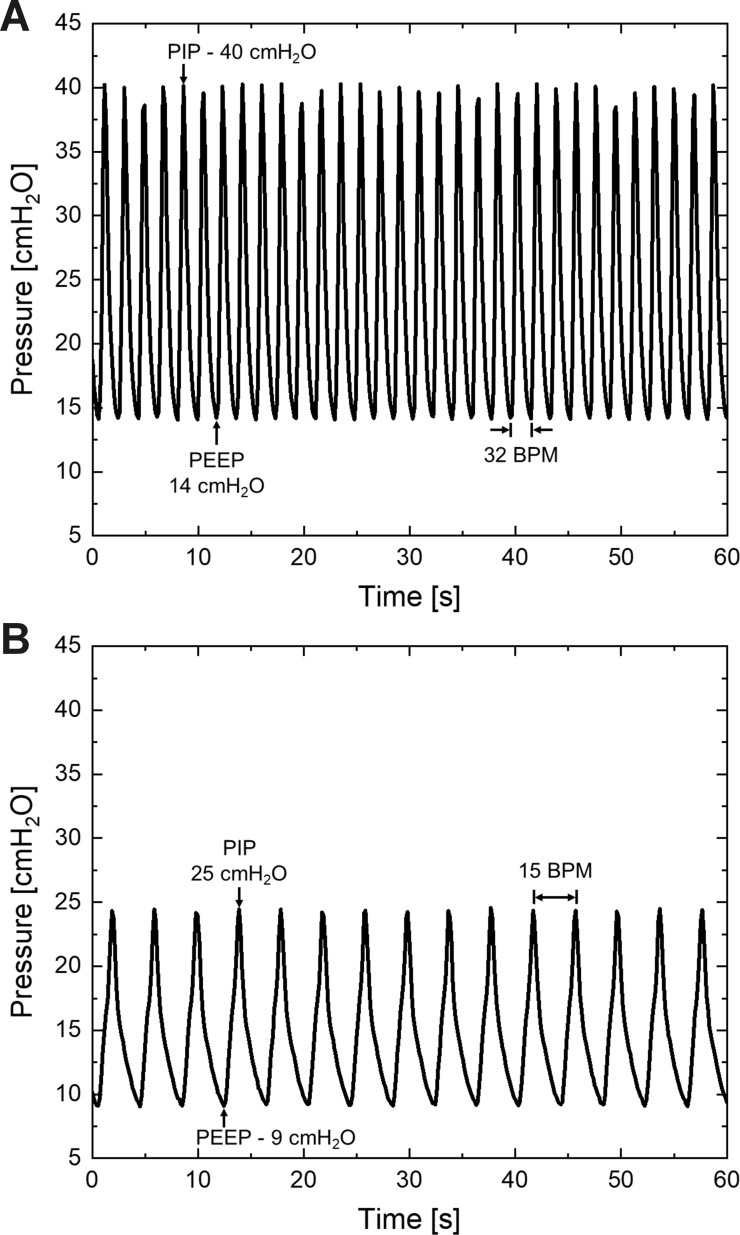
Performance of the RapidVent during simulation of its use in various clinical scenarios. (A) Pressure versus time when the ventilator operates at PIP of 40 cm-H_2_O and 32 BPM, and (B) PIP of 25 cm-H_2_O at 15 BPM.

We measured the device performance under conditions simulating its use in patients at various treatment stages (Fig 6). To mimic the use of the device for sick patients, we tested the device at high PIP / PEEP of 40 / 14 cm-H_2_O. At these settings, the mechanical ventilation rate is 32 BPM and the peak flow is steady at 32 L/min. To mimic the use of the device for patients needing lower PEEP, we tested the device at low PIP / PEEP settings of 25 / 9 cm-H_2_O. At this low setting, the ventilation rate is 15 BPM and the peak flow rate is 15 L/min.

The durability of the RapidVent prototypes was tested by running 10 devices for 84 hours, equivalent to about 100,000 breathing cycles per device without interruption. Across all devices tested, including the durability study and the animal study described below, the devices combined ran over two million breathing cycles without failure. We also tested the performance across 22 prototypes. All 22 devices were able to achieve a PIP of 30 cm-H2O, a flow rate of 40 L/min, and 10 BPM. The supporting information shows additional test results (S3 Fig) and durability testing (S4 Fig).

### Animal testing

**Fig 7:**
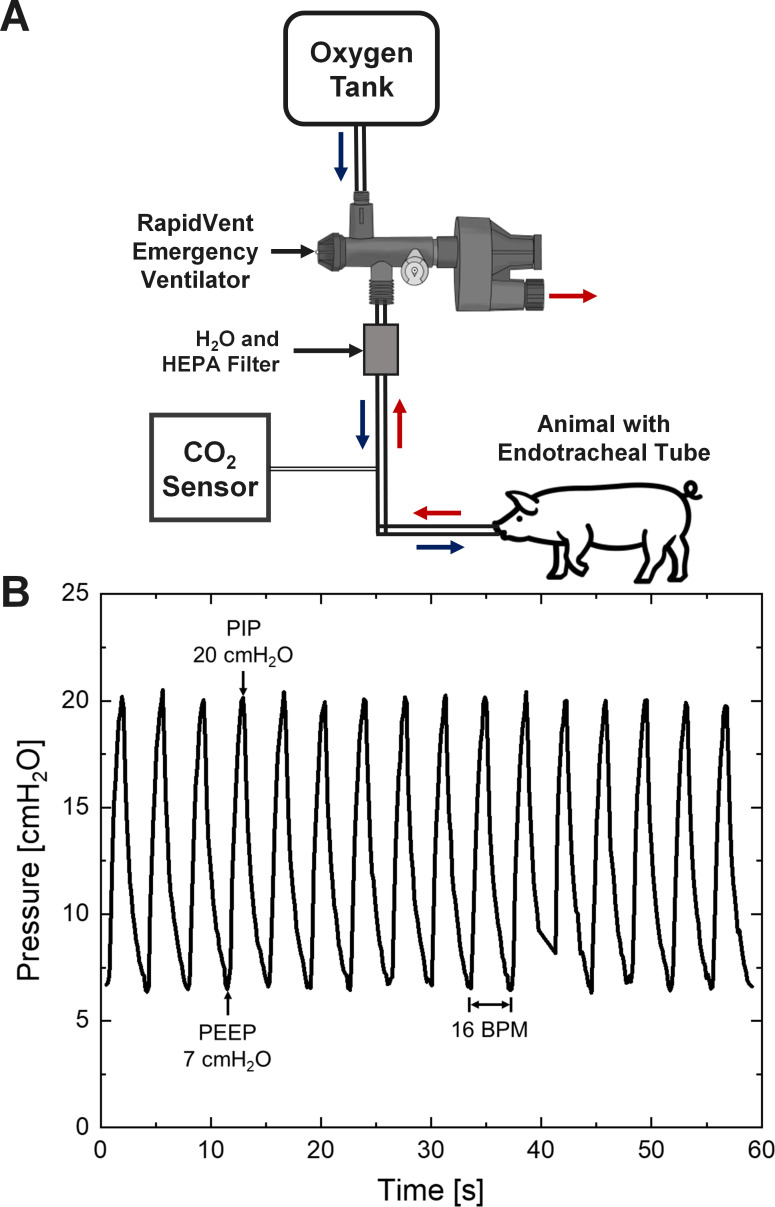
Animal testing. (A) Schematic of the setup used during the mechanical ventilation of sedated pigs. (B) Example of the breath cycles of the animal induced by the RapidVent when the PIP is set to 20 cm-H_2_O at 16 BPM.

Three animal tests were conducted to validate the EV and to evaluate the potential for use in humans infected with COVID-19. The animal study was approved by the University of Illinois Institutional Animal Care and Use Committee (IACUC) which supervises and ensures the ethical and responsible treatment of animals. The supporting information describes test methods and results (S1 Appendix). The animals were Yorkshire and Yorkshire cross pigs (200–280 lbs.). The pigs were sedated, intubated, and their body temperature, heart rate and breathing rate were continuously monitored as shown in Fig 7. Venous blood samples (1 ml/sample) were taken periodically and assayed for blood pH, partial pressure of oxygen, and partial pressure of carbon dioxide using a portable handheld blood gas analyzer (i-STAT, Abbott,) and end tidal CO_2_ was monitored using a portable electronic capnograph (N-85 Nellcor, Coviden).

The first animal test lasted three hours and the second animal test lasted 24 hours. The supplementary information shows example data and comparison with a commercially available EV (S5 and S6 Figs). In both tests, the animal was supplied with pure oxygen through the RapidVent. Fig 7 shows pressure and volume data for the RapidVent EV during the first animal test. In the second animal test, multiple pigs were cycled through every 3–12 hours, as the pigs were removed from the study when their blood pH continuously dropped below the acceptable range (7.3–7.5). The team hypothesized that the dead space within the long tube between the EV and the animal (> 1.5 m) limited the ventilation, resulting in respiratory acidosis.

The third and final animal test of duration four hours was conducted with a short tube between EV and the animal (< 0.2 m). Adjustments to the respiration rate allowed the team to increase or decrease CO_2_ in the animal’s blood, indicating that the EV could in fact control ventilation of the animal. To simulate abnormal respiratory mechanics, a 40 lb. sandbag was placed on the animal, resulting in a decrease in PEEP and PIP. By adjusting the PIP setting on the RapidVent, the pressure levels returned to their original set points within a few seconds (S7 Fig). At the beginning of the test when the animal was deeply sedated, there was no spontaneous breathing and the pressure was very stable. Later in the study, the animal began to spontaneously breathe and the pressure pattern changed. To compensate for the spontaneous breathing, the rate dial was adjusted to fully open to allow more ventilation and the pressure pattern was restored. This result indicates that a human may be able to breathe with help from the EV when sedated and may also be able to comfortably breathe when less sedated and spontaneously breathing. The RapidVent was very stable during the animal tests, with little fluctuation in cycle time over the course of the tests (S8 Fig).

### Electronic monitoring system

Hospital-grade ventilators have integrated sensors that monitor patient breathing and ventilator operation, and provide information to the caregiver about PIP, PEEP, respiratory rate, and other clinical data. They also trigger alarms when the ventilator stops working or when the breathing pattern changes in a way that requires attention. We developed a simple and low-cost electronic monitoring system for use with the RapidVent. The device uses a pressure sensor, microcontroller, and signal processing algorithms to calculate PIP, PEEP, and respiratory rate and to sound an alarm when a patient stops breathing or the ventilator stops working. The alarm is a loud buzzer. The monitoring system does not measure tidal volume or oxygen concentration, as these measurements would require additional sensors. This section briefly describes the alarm concept and its integration with RapidVent. A study on the algorithm development and mechatronic system design was published in a separate article [42].

**Fig 8:**
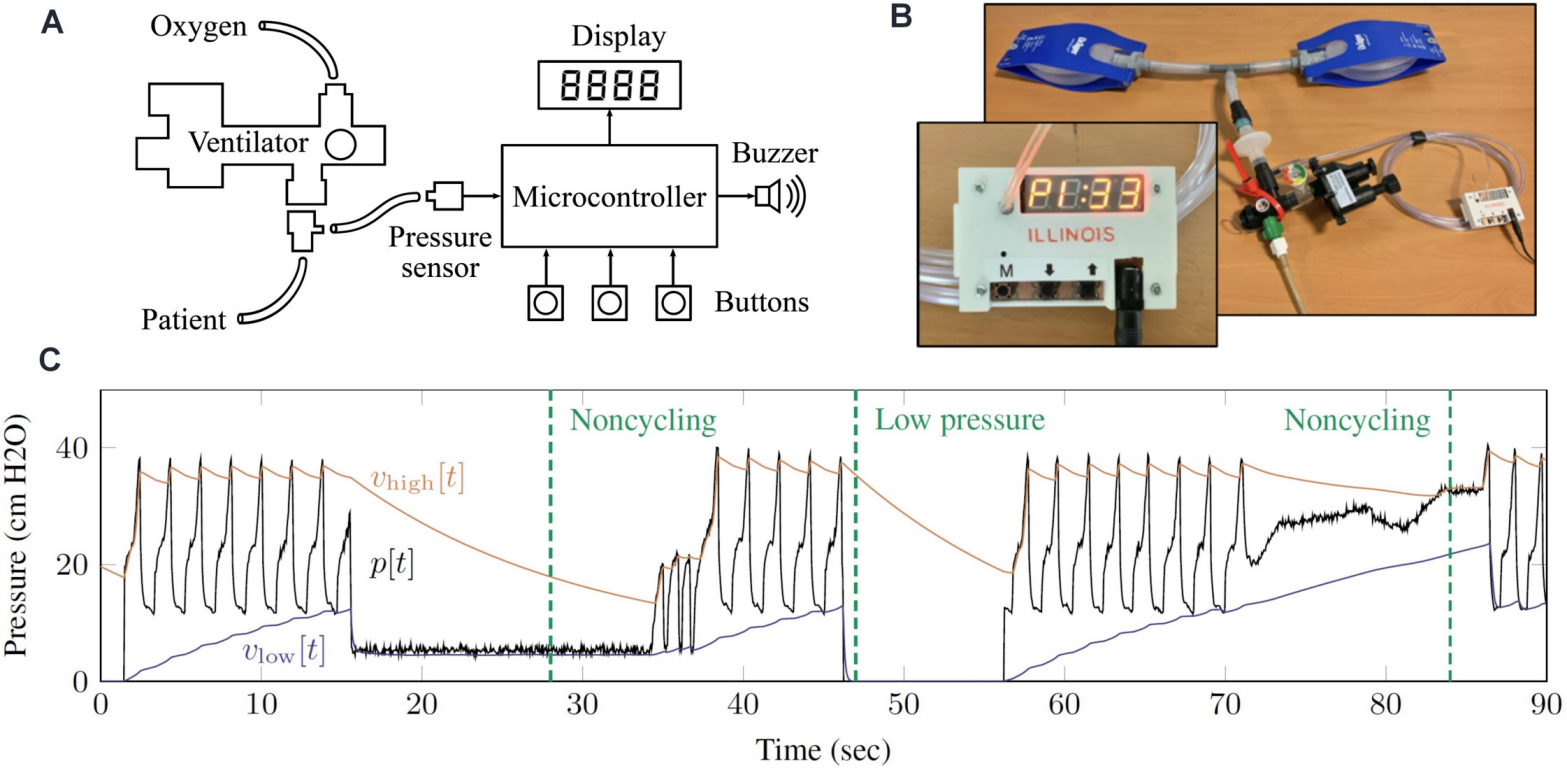
Overview of electronic monitoring system. (A) System diagram showing pressure sensor input from RapidVent. (B) Photograph of alarm with display connected to Drager test lungs. (C) Data from Drager test lung showing different alarm conditions. The measured pressure is *p* and the envelopes *v*_high_ and *v*_low_ are used to track the breath cycle. The noncycling alarm is triggered when the pressure envelopes are too close together.

Fig 8 shows the alarm and example data. An electronic pressure sensor measures pressure on the patient side of the ventilator circuit, and the senor data is sampled 100 times per second by a microcontroller. The signal processing algorithm applies a pair of nonlinear recursive envelope trackers to follow the maxima and minima of the pressure signal across breath cycles. A signal processing algorithm converts the pressure measurement *p* into PEEP, PIP, and breathing rate which are displayed on the front of the device. When the high pressure and low pressure are too close together, the system triggers an alarm. Alarms are also triggered when the PEEP, PIP, or respiratory rate fall outside a normal range, which can be specified by the user with a set of buttons on the front of the device.

The alarm functions of the electronic monitoring system were validated using data from the animal tests as well as real-time testing using a Drager test lung. First, the alarm algorithm was validated on pressure data that was previously collected during animal testing with the RapidVent, which included several alarm-triggering events. When the animal began to spontaneously breathe, causing the pressure during inhalation to drop near atmospheric pressure, the low-pressure alarm triggered to alert the clinician that the patient is attempting to breathe. In another event, the animal rolled over and disconnected the respiratory circuit, which triggered the noncycling alarm. The noncycling alarm was also triggered when the exit port was blocked for a few seconds to measure tidal volume. Following development and validation using data from the animal tests, the algorithm was then implemented on a prototype monitoring device and tested using a test lung, which was deliberately manipulated to trigger alarms. Fig 8C shows example data. The algorithm instantly detects a disconnection event that causes pressure to drop to zero. It also correctly detects noncycling conditions, even when the pressure signal remains in a normal range, because the high- and low-pressure envelopes converge.

## Discussion

The RapidVent performance compares well with the AARC recommendations for a low function mechanical ventilator. Specifically, the AARC recommends that a ventilator must be able to provide a PEEP of 10–20 cm H_2_O, tidal volume of at least 300–600 mL and minute ventilation of 10–15 L/min [12]. The RapidVent is capable of PEEP up to 15 cm H_2_O, which is somewhat higher than other EVs. Functional testing on the test lung showed tidal volume 500 mL and minute ventilation in the range 8–16 L/min. The AARC also recommends electronic monitoring and alarms capable of detecting disconnect, apnea, high pressure, and low pressure, which are all within the capability of the electronic monitoring system discussed here. We specifically chose a gas-powered pressure-switched EV rather than a ventilator design with even lower function. There has recently been a proliferation of very low function ventilator designs [43]. Some very low function devices are based on bag-squeezing mechanisms are unable to sustain a positive PEEP and do not meet the minimum recommendations of AARC. The training and expertise of the EV user is important as well. The RapidVent was designed according to the needs of critical care physicians and respiratory therapists, and we envision that a highly trained person would supervise its use.

While the results described here are promising, additional work is required before such a device could be deployed in a clinical setting. The next steps could include additional tests on animals or humans. Regulatory approval is required before a medical product can be used in a clinical application. We specifically chose to not pursue clinical testing or regulatory approval, assuming that these advancements would be more effective if done by a commercial organization. Thus, we made the technology available through a free license [44] and an open source license for the alarms [45]. More than 70 organizations have accessed the technology through the free license, including organizations in the following countries: Argentina, Canada, Egypt, France, India, Korea, Mexico, Philippines, South Africa, Spain, Syria, Taiwan, Turkey, and the United States. Many of these groups have made working prototypes and two groups have announced mass production of an emergency ventilator derived from the RapidVent [46, 47].

The gas source is a key issue for a gas-powered ventilator. The RapidVent is designed for use with 50 psig gas, which could by pure oxygen, air, or a gas mixture. When connected to oxygen, the RapidVent can deliver either 100% oxygen or 50% oxygen using an entrainment nozzle. There are a variety of commercially available entrainment nozzles that could be used to set the oxygen mix at a different ratio. While 50 psig oxygen is widely available in North America and some other parts of the world, some regions of the world use hospital oxygen or air supply at a lower pressure. The RapidVent can easily be modified for a different pressure setting, by tuning the compliance of the diaphragm and the spring constant of the integrated spring.

The ventilators in this study were produced using additive manufacturing, which allows for rapid prototyping and low volume production. The mechanical designs are amenable to injection molding which would be appropriate for higher volume production. Injection molding however requires a resource investment for tooling and takes somewhat longer to scale up compared to additive manufacturing which can begin quickly. We estimate the cost to produce RapidVent to be around $100 per device for volume in the tens of thousands of devices. A key cost of producing medical devices is the quality system that ensures device safety.

The methods described in this study could help other teams rapidly develop new products in response to an emergency. The product development process employed here was like those used for conventional research projects, however some specific modifications were made for the emergency. During the design iteration phase, many prototype component designs were additively manufactured with the understanding that most of them would be thrown away after testing. While this approach generated extra waste, it significantly accelerated the project. The ability to prototype using AM of functional engineering materials also accelerated the project, as the transition from development to functional testing was nearly seamless. Finally, while product development teams are normally organized by function and expertise, the need for rapid progress made it necessary to work in interdisciplinary teams to solve both component level and system level problems in parallel.

## Conclusion

In response to the COVID-19 pandemic, we designed, prototyped, and tested an emergency ventilator. Additive manufacturing in durable end-use materials enabled ultra-fast development of the product through rapid design iterations, functional testing, and animal testing. An electronic monitoring system provides information about EV operation and provides an alarm when the device fails or patient breathing stops. To our knowledge, this is the first article to report detailed mechanical design, functional testing, durability testing, animal testing, and integrated electronic monitoring with an emergency ventilator. The product design, user manual, and training materials are available through a free license that has been accessed by more than 70 organizations in 15 countries, and the electronic monitoring system is available through an open-source license. More than 50 people participated in the project and worked about 5,000 hours over the 19-day project period. The team consisted of engineers, scientists, physicians, designers, and animal scientists.

## Supporting information

S1 Appendix Animal testing methods and results. (PDF)

S1 Fig RadpidVent bill of materials. (TIF)

S2 Fig Exploded view of RapidVent CAD with components labeled. (TIF)

S3 Fig The durability of the RapidVent prototypes was tested by running 10 devices for 84 hours. For the first 72 hours, the devices were cycled under a controlled inlet flow rate of 20 L/min. During this period, no failures were observed. This was important for the team to assure the ability of the mechanical components of the design to resist any fatigue-induced failure for three days uninterrupted. After the first three days, half of the ventilators were adjusted to 40–45 cm-H2O PIP and 30 BPM, while the rest were tested at 25 cm-H2O PIP at 15 BPM. The high pressure and rate correspond to conditions associated with a very sick COVID-19 patient, while the lower pressure and rate correspond to a patient that is less sick.
(TIF)

S4 Fig Performance comparison of 22 RapidVent prototypes. (A) The rate dial of each ventilator is set to 10 BPM, then the PIP dial is set to the maximum setting. For each device, the bar shows the value of maximum flow rate at the PIP point, and the circular marker shows the corresponding value of the PIP. (B) The rate dial of each ventilator is set to 10 BPM, then the PIP dial is set to 30 cm-H2O.
(TIF)

S5 Fig Oxygen pressure and flow levels during the initial 3-hour ventilation test. A: RapidVent prototype under test. B: Reference design.
(TIF)

S6 Fig Oxygen pressure and flow levels during the 24-hour ventilation testing. A: Animal 1 on RapidVent prototype 1 around mid-point. B: Animal 2 on RapidVent prototype 1 around mid-point. C: Animal 3 on RapidVent prototype I around mid-point. D: Animal 1 on RapidVent prototype II around mid-point. E: Animal 2 on RapidVent prototype 2 around mid-point.
(TIF)

S7 Fig Pressure data acquired from the patient port of the RapidVent prototype with minimal circuit during 4 hour test taken. A: at test beginning. B: during the time where the animal was spontaneously breathing. C: where the rate dial was adjusted to fully open to allow more ventilation. Note the shape of the pressure curve restores to the shape observed in A, where the device was supporting the breathing. D: when 40 lbs. of sandbags were placed on the animal’s ribcage simulating restricted breathing. The pressure drops almost immediately in reaction to the available volume being restricted due to the weight. The ventilator was adjusted by increasing the PIP dial to compensate for the burden, and the settings stabilized in approximately 15 seconds after adjusting.
(TIF)

S8 Fig Poincaré plot of the PIP and PEEP set point variations during animal tests. (A) Scatter plot of PIP at cycle [n] versus PIP at cycle [n+7] to observe the longitudinal variations of pressure during the testing. The PIP mean ± standard deviation is 17.25 ± 0.53 cm-H2O and the covariance [n, n+7] is 0.16. (B) Scatter plot of PEEP at cycle [n] versus PEEP at cycle [n+7] to observe the longitudinal variations of pressure during the testing. The PEEP mean ± standard deviation is 8.17 ± 0.3 cm-H2O and the covariance [n, n+7] is 0.06.
(TIF)

S1 File (PDF)
