# Supplementary material for: Emergency ventilator for COVID-19
Source: PLoS One. 2020 Dec 30;15(12):e0244963. doi: 10.1371/journal.pone.0244963 (PMC7773325; doi:10.1371/journal.pone.0244963)
Supplement: S1 Appendix — (PDF) [file pone.0244963.s001.pdf]

Appendix: Animal testing methods and results

Objective

The objective of the animal studies described were to test and validate the use of a rapid prototyped emergency gas powered ventilator in pigs that is designed after an approved, commercially available ventilator for potential use in humans infected with COVID-19 virus. The design, development, and manufacture of an emergency gas powered respirator/ventilator that could help alleviate the anticipated shortage here in our local community (and potentially more broadly) provided strong motivation to the team to investigate the performance of the device quickly and thoroughly. This animal study represented a critical testing step to build confidence in the design and ultimately support the effort to explore approval for this ventilator for human use. Since the end goal was for these ventilators to be used in human patients, pigs were of special interest because the size of their lungs is comparable to that of humans.

Materials and methods

Yorkshire and Yorkshire cross pigs (200-280 lbs) were used to test the performance of RapidVent ventilator. The animal study was approved by the University of Illinois Institutional Animal Care and Use Committee (IACUC) which supervises and ensures the ethical and responsible treatment of animals. Animals were used under a protocol (# 20071) approved by the IACUC. Pigs were moved from the farm to the Physiology Research Laboratory at the University of Illinois Urbana-Champaign on the day prior to the scheduled ventilation study. All pigs had feed withheld overnight prior to ventilation procedures but the pigs had access to water.

Animal sedation and intubation

All pigs received a sedative cocktail (TARK) consisting of Telazol (tiletamine and zolazepam; Pfizer, New York, NY), Atropine (Neogen Corporation, Lexington, KY), Rompun (xylazine; Lloyd Laboratories, Shenandoah, IA) and Ketamine (Ketaset, Fort Dodge Animal Health, Fort Dodge, IA) intramuscularly, then again intravenously via the ear vein canula, as necessary. We used an endoscope fitted with a large animal blade to place the endotracheal tube into the trachea. The animals sedated as described, were placed in sternal recumbency and had the endotracheal tube inserted into the trachea. The balloon was inflated with a 5-12cc syringe, depending on the size of the balloon, in order to fix the endotracheal tube in the trachea. Endotracheal administration of 3-5% isoflurane was administered in oxygen as necessary during the 24 hour ventilation test.

Animal monitoring

During the course of the testing, multiple parameters were monitored including body temperature, heart rate and breathing rate as well the palpebral reflex to assure the appropriate depth of anesthesia. Blood pressure was monitored with a Cardell® pressure monitor. Briefly, a blood pressure cuff was placed around a front limb to obtain indirect measurements of blood pressure. Measurements were taken every 15 minutes. Breathing rate was constantly monitored. Venous blood samples (1ml/sample) were taken periodically and assayed for blood pH, partial pressure of oxygen, and partial

pressure of carbon dioxide using a portable handheld blood gas analyzer (i-STAT, Abbott, Chicago, IL) and end tidal CO_2 was monitored using a portable electronic capnograph (N-85, Nellcor, Coviden/Medtronic,Minneapolis, MN).

Anesthesia during ventilation testing

Once the animal was sedated with TARK, it was placed on isoflurane (to effect) via an endotracheal tube placed in the trachea of the pig to administer inhalation anesthesia (24 hour test). Alternatively, after sedation the pigs were maintained on intravenous anesthesia (3 and 4 hour tests) with combinations of Dexmedetomidine (Dexmedesed®, 1 mcg/kg/hr CRI IV, Dechra Vet Products, Overland Park, KS), Ketamine (Ketaset®, 1-10mg/kg/hr CRI IV, Fort Dodge Animal Health, Fort Dodge, IA) or Propofol (Propothesia®, 10-20mg/kg/hr CRI IV Covetrus, Indianapolis, IN). Anesthetic medications will be selected based on animal condition to maintain a stable plane of anesthesia. Dexmedatomidine anesthesia was reversed by administration of Antisedan (Zoetis US, Parsippany, NJ).

Simulated labored breathing

To simulate labored breathing, a 40 pound sandbag was placed on the animals' ribcage while in lateral recumbency. The time period examined was 30 minutes to 1 hour depending on the study.

Post-study animal recovery

After the ventilation testing was completed, all animals on the ventilator were returned to a clean, dry pen with a rubber floor mat in a heated recovery area to recover. Rectal temperatures and heart rate were taken every 15 minutes until the animals were sternal. Animals remained in the recovery facility for 24 hours and after fully regaining the ability to walk normally they were returned to their pens in the Biomedical Housing Area at the facility. The pigs were housed individually following surgery until recovery at 2 days, but were able to have fence line contact with other pigs. Antibiotic therapy (Excede, Pfizer, New York, NY) at 5cc/100 kg (5.0 mg ceftiofur equivalent/kg)) was administered after the ventilation procedure and at weekly intervals for 1 week as needed. The following parameters were: temperature, appetite, behavior and tenderness at the intubation site. If there were any deviations from normal values in these parameters, the veterinary staff was consulted for appropriate treatment. We watched for vomiting and ataxia. Supplemental heat and warming was provided if necessary. Complete medical records were kept on each animal.

Initial animal testing of the RapidVent (3 hours) with sensor monitoring station inline before the ventilator

An initial study was performed with a single animal to test that the ventilator was functioning properly before investing in a long term (24 hr) test. This test lasted for 3 hours using 2 of the prototype RapidVent ventilators and a comparable commercially available Vortran model 5011 emergency gas powered ventilator each for approximately 1 hr. During the last hour of the second RapidVent testing, a 40 pound sandbag was placed on the ribcage of the anesthetized animal to simulate labored breathing. During this test the RapidVent was attached to monitoring electronic equipment that included mass flow,

pressure, and temperature of the gas exchanged to the animal. Approximately 1.6 m of 22 mm inner diameter corrugated tubing connected the device under test to the animal. This test was performed as an equivalency test to determine if the prototype ventilator was substantially different from the commercially available ventilator. The parameters that were collected on the ventilator (mass flow rate, pressure, temperature) were used to evaluate cycling performance and durability of the parts after animal testing.

Long-term animal testing of the RapidVent (24 hours) with sensor monitoring station inline before the ventilator

The full 24 hour tests required the device to run continuously for 24 hours. The parameters that were collected on the ventilator included mass flow rate, pressure, pCO_2, and were used to evaluate cycling performance and durability of the parts after animal testing. The pigs were changed every 3-12 hours as necessary so that a single ventilator was used continuously for 24 hours. Two RapidVent prototypes were tested with the same circuit as for the three hour test for 24 hours and a total of five pigs were used to complete this study.

Short-term animal testing of the Rapidvent (4 hours) with sensor monitoring station inline after the ventilator

The final study was performed with a single animal to test that the ventilator functioned properly when placed directly before the endotracheal tube placed into the pig, as it would be in a human patient, greatly reducing the dead volume of the circuit. This test was run for 4 hours using 1 of the prototype RapidVent ventilators. During the last 30 minutes of the testing, a 40-pound sandbag was placed on the ribcage of the anesthetized animal to simulate labored breathing. During this test the device was instrumented only with pressure sensing on the animal side of the device and the exhalation port of RapidVent was intermittently attached to a Wright spirometer and electronic mass flowmeter to evaluate tidal volume. Additional monitoring electronic equipment included end tidal CO_2. This test was performed to determine if the prototype ventilator, when placed as it would be in a human patient, would oxygenate and ventilate the animal. Further, this test investigated whether adjusting the flow and pressures through the device could maintain and/or adjust the acid-base balance of the blood around physiological pH limits of 7.4. The ability to control blood acidosis and alkalosis of the animal was a critical parameter that needed testing. Cycling performance and durability of the parts were compared to prior results to confirm the device was operating as intended after animal testing.

Results

Test 1: Three-hour test of the RapidVent ventilator

Animal monitoring was performed, as described above, every 15 minutes during the 3 hour test of the RapidVent ventilator. The temperature of the pig remained constant throughout the test. The heart rate of the pig decreased (evidence of the sedation level and relaxation of the animal), and the oxygen saturation increased as the test progressed, showing there was adequate oxygenation by the ventilator. The ventilator was only adjusted to maintain a peak inspiratory pressure (PIP) of ~20 cmH_2O, and a reasonable breathing rate (respiratory rate) between 12 and 20 breaths per minute. Post procedural temperature, appetite, and behavior were observed to be in the normal ranges (body temperature = >98°F, mucous membranes = pink, not pale, white, gray, or blue, respiratory rate (breaths/minute) = 6-20, heart rate (beats/minute) = 60-140) and no tenderness at the intubation site was observed.

Test 2: Twenty-four-hour testing of the RapidVent ventilator

Animal monitoring was performed, as described above, every 15 minutes during the 24 hour test of the RapidVent ventilator. The temperature of each pig stayed constant with supplemental heat. Oxygen saturation levels remained constant within the acceptable range (>95%) on all pigs. The heart rate of each pig was monitored and anesthesia was adjusted according to heart rate and previously described parameters The flow rate and the pressure of the oxygen flow were monitored over the 24 hour test period. The results show that both the rate and pressure was relatively constant over the test period. Pigs were removed from the trial when their blood pH continuously dropped below the acceptable range (7.3-7.5). The ventilators were adjusted in response to respiratory acidosis, primarily in an attempt to increase ventilation (increase PIP pressure, increase respiratory rate). However, the pH of the blood continued to be below acceptable ranges even with administration of different therapies including administration of sodium bicarbonate, changing the IV fluids to Vetivex electrolytes and adjustments in the anesthesia. The devices were also checked after each oxygen tank change to ensure consistent settings were achieved throughout. Post procedural temperature, appetite, and behavior were observed to be normal (see ranges above) and no tenderness at the intubation site was observed for each of the 5 pigs.

Test 3: Four-hour test of the RapidVent ventilator

Animal monitoring was performed, as described above, every 15 minutes during the 4 hour test of the RapidVent ventilator. The animal's heart rate, temperature, and oxygen saturation remained constant (>95%) throughout the test when measured by a pulse oximeter. Blood pressure readings performed approximately every 30 minutes also remained within a normal range of >60 mm Hg (mean) and >90 mm Hg (systolic). With the greatly reduced dead volume in the respiration circuit, ventilation improved dramatically >60 mm Hg (diastolic) and much more control was achieved over the respiratory physiology using the ventilator. Changes to the respirator rate for a constant PIP setting demonstrated an ability to tune the end tidal and blood gas readings more alkalotic or acidotic on command. This control was sensitive; around ¼ turn of the

respirator rate /rate dial on the ventilator affected the end tidal CO_2 readings +/- 4-6 points from a stable 48.

Pressure data was acquired from the patient port of the RapidVent prototype with a minimal circuit during 4 hour test. At the beginning when the pig was deeply sedated there was no spontaneous breathing and the pressure was very stable. When the sedation became lighter the animal began to spontaneously breath and the pressure pattern changed. To compensate for the spontaneous breathing, the rate dial was adjusted to fully open to allow more ventilation. Note the shape of the pressure curve restores to the shape observed in, where the device was supporting the breathing. Near the end of the test period (the last 30 minutes) we placed a 40 lb sandbag on the animal's ribcage, simulating restricted labored breathing with reduce inspiratory volume." The pressure droped almost immediately in reaction to the available volume being restricted due to the weight. The ventilator was adjusted by increasing the PIP dial to compensate for the burden, and the settings stabilized in approximately 15 seconds after adjustment.

End tidal CO_2 was measured with a portable meter (N-85, Nellcor, Coviden/Medtronic, Minneapolis, MN). The pCO_2 levels were measured from a blood sample using a portable blood gas analyzer (i-STAT, Abbott, Chicago, IL). The pH was also measured by the i-STAT portable blood gas analyzer. At approximately 1.5 hours into the test, the bypass hole in the modulator of the ventilator was plugged in order to take Wright spirometer readings. The rise in CO_2 correlated well with the drop in pH, and triggered spontaneous breathing. Around hour 2, the rate dial was adjusted fully open to allow more ventilation, restoring supported breathing and correcting CO_2 and pH.

Post procedural temperature, appetite, and behavior were observed to be normal (see ranges above) and no tenderness at the intubation site was observed.

RapidVent stability during animal tests

The RapidVent performance was extremely stable in all the tests. S9 Fig shows a Poincaré recurrence plot of the PEEP and the PIP over one hour of breathing cycles during the 24-hour animal test. The figure shows PEEP and PIP values and their correlation with the PEEP and PIP values separated by seven cycles. The data show a single, narrow cluster, indicative of stable EV performance.
