# Supplementary figures and images for: Emergency ventilator for COVID-19
Source: PLoS One. 2020 Dec 30;15(12):e0244963. doi: 10.1371/journal.pone.0244963 (PMC7773325; doi:10.1371/journal.pone.0244963)

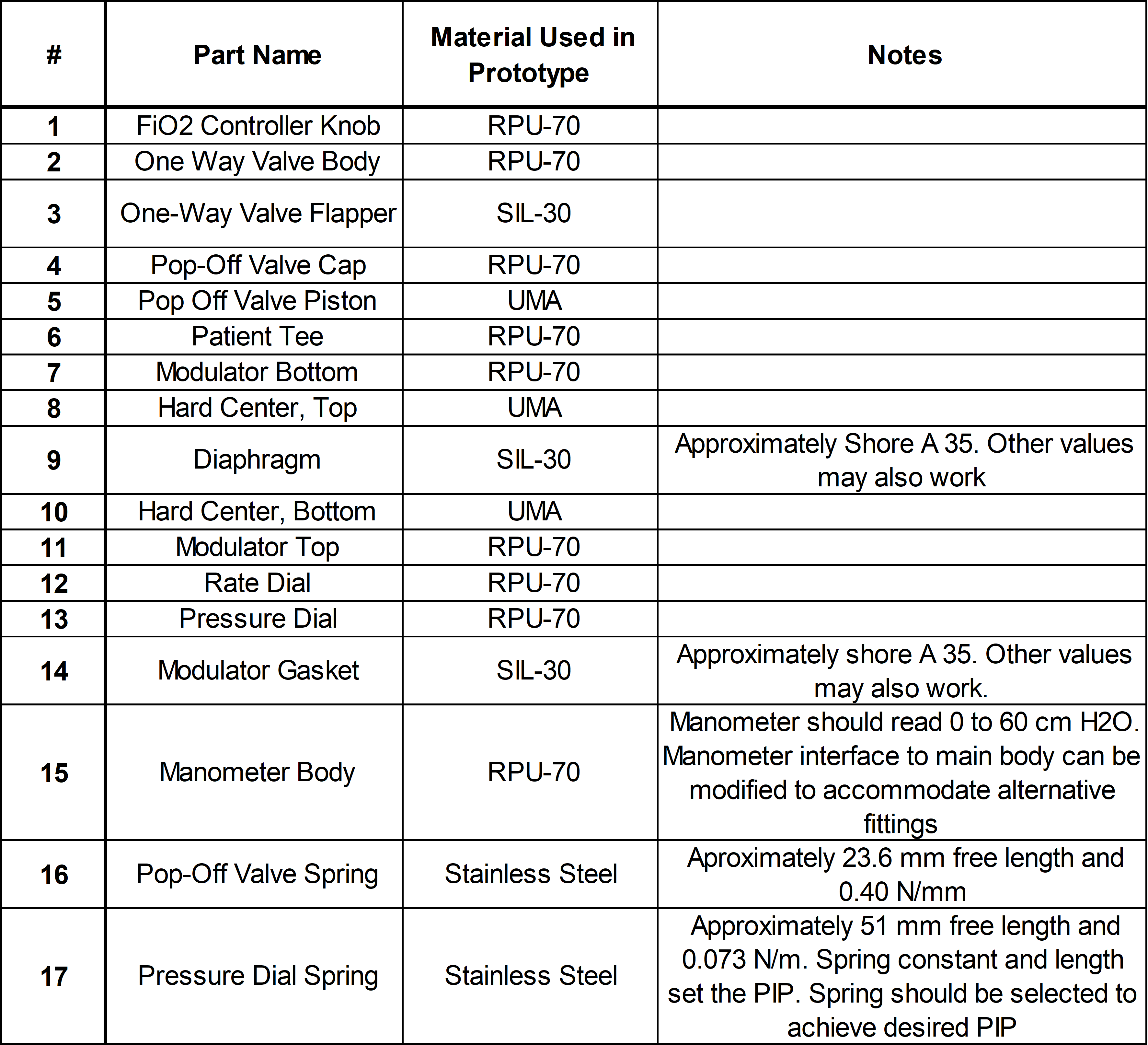

Supplement: S1 Fig — (TIF) [file pone.0244963.s002.tif]

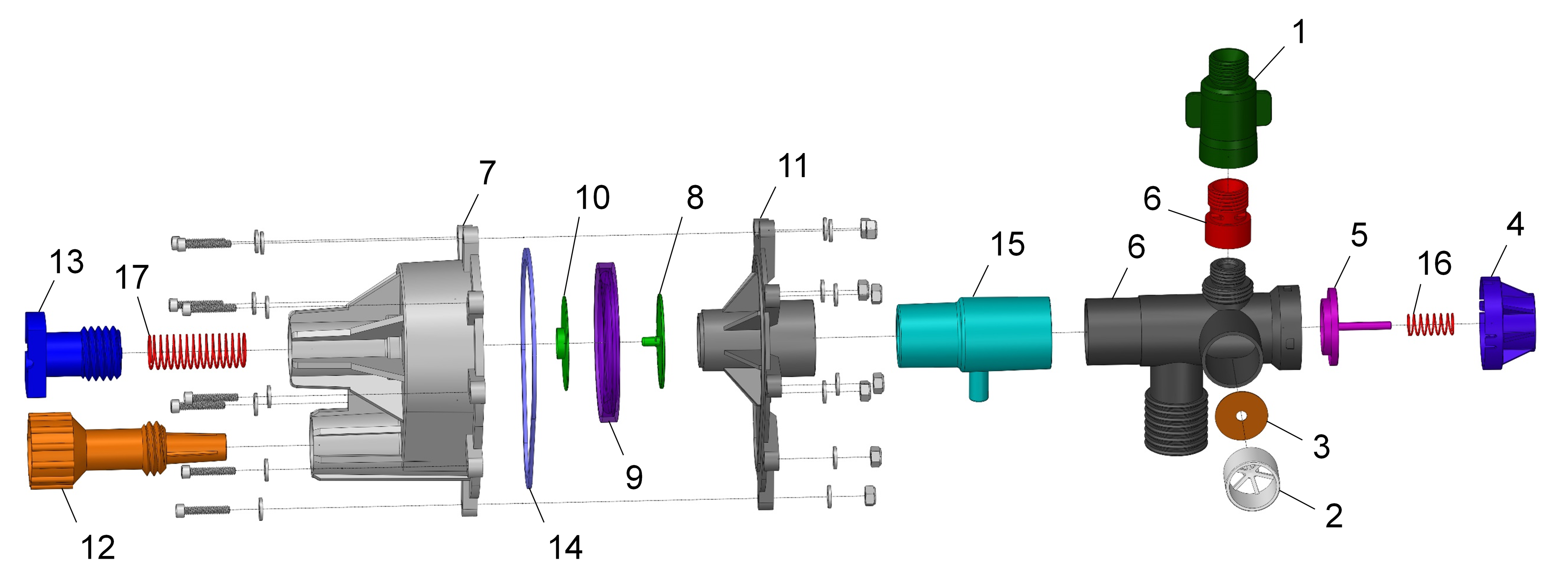

Supplement: S2 Fig — (TIF) [file pone.0244963.s003.tif]

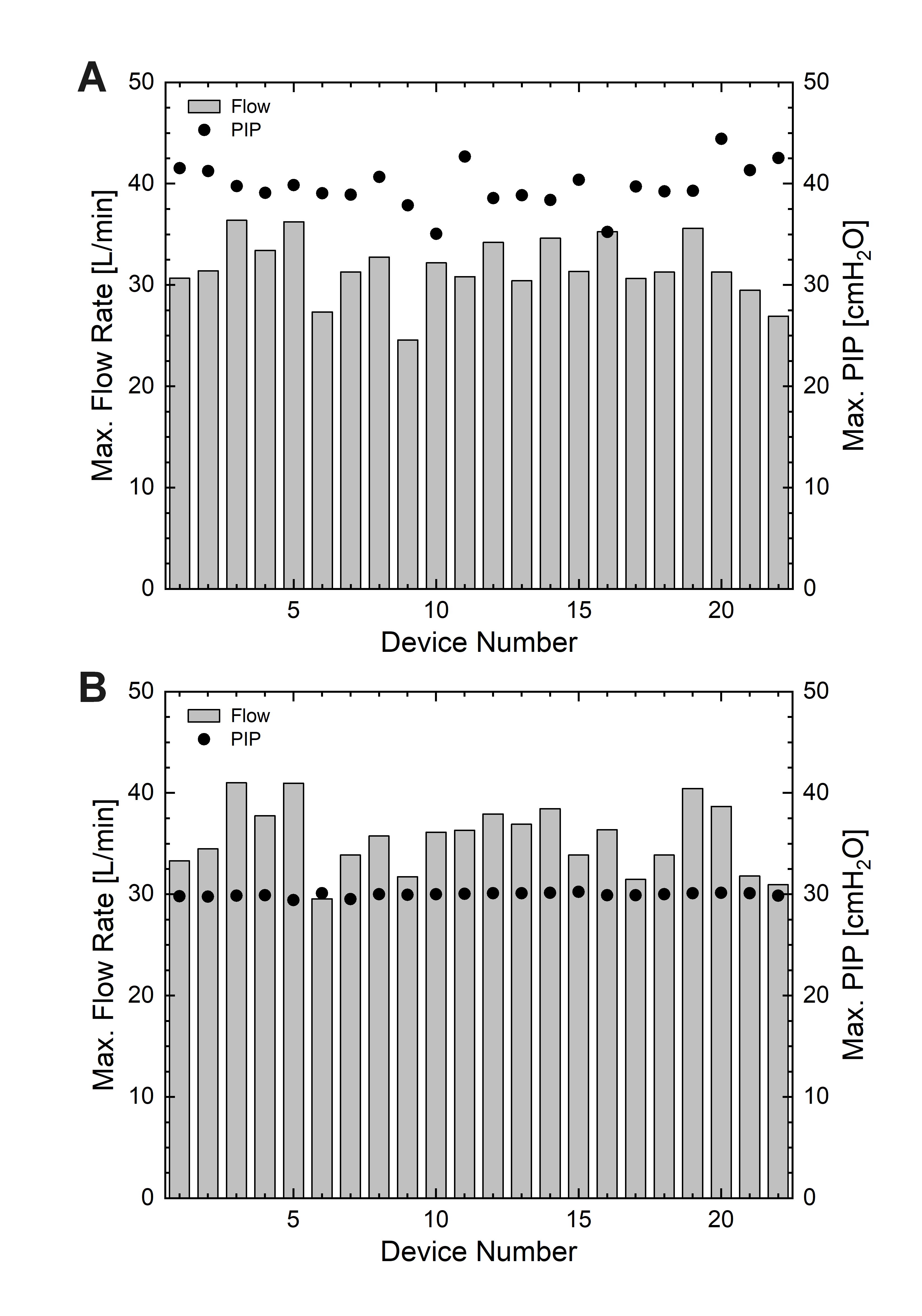

Supplement: S3 Fig — For the first 72 hours, the devices were cycled under a controlled inlet flow rate of 20 L/min. During this period, no failures were observed. This was important for the team to assure the ability of the mechanical components of the design to resist any fatigue-induced failure for three days uninterrupted. After the first three days, half of the ventilators were adjusted to 40–45 cm-H2O PIP and 30 BPM, while the rest were tested at 25 cm-H2O PIP at 15 BPM. The high pressure and rate correspond to conditions associated with a very sick COVID-19 patient, while the lower pressure and rate correspond to a patient that is less sick. (TIF) [file pone.0244963.s004.tif]

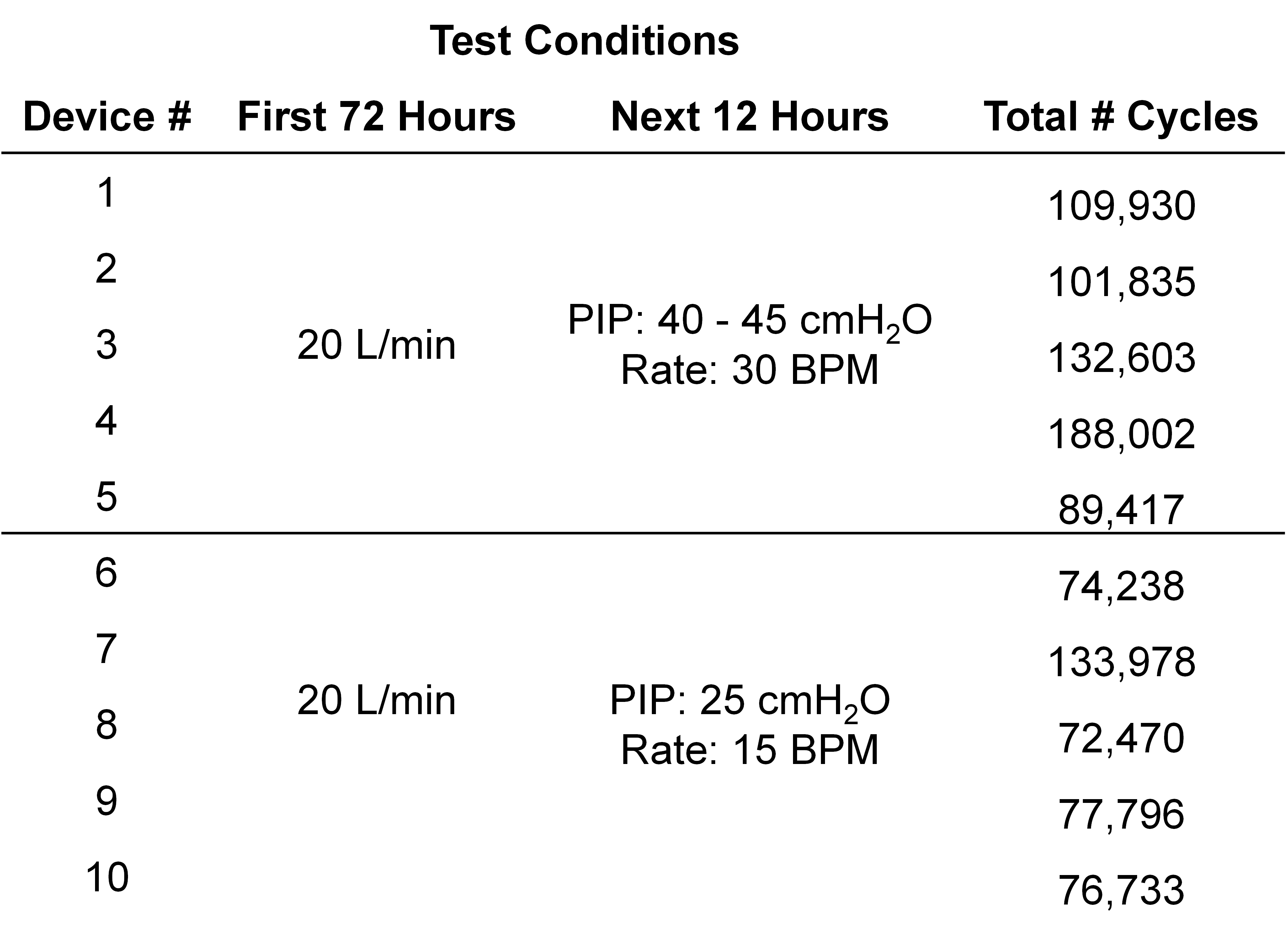

Supplement: S4 Fig — (A) The rate dial of each ventilator is set to 10 BPM, then the PIP dial is set to the maximum setting. For each device, the bar shows the value of maximum flow rate at the PIP point, and the circular marker shows the corresponding value of the PIP. (B) The rate dial of each ventilator is set to 10 BPM, then the PIP dial is set to 30 cm-H2O. (TIF) [file pone.0244963.s005.tif]

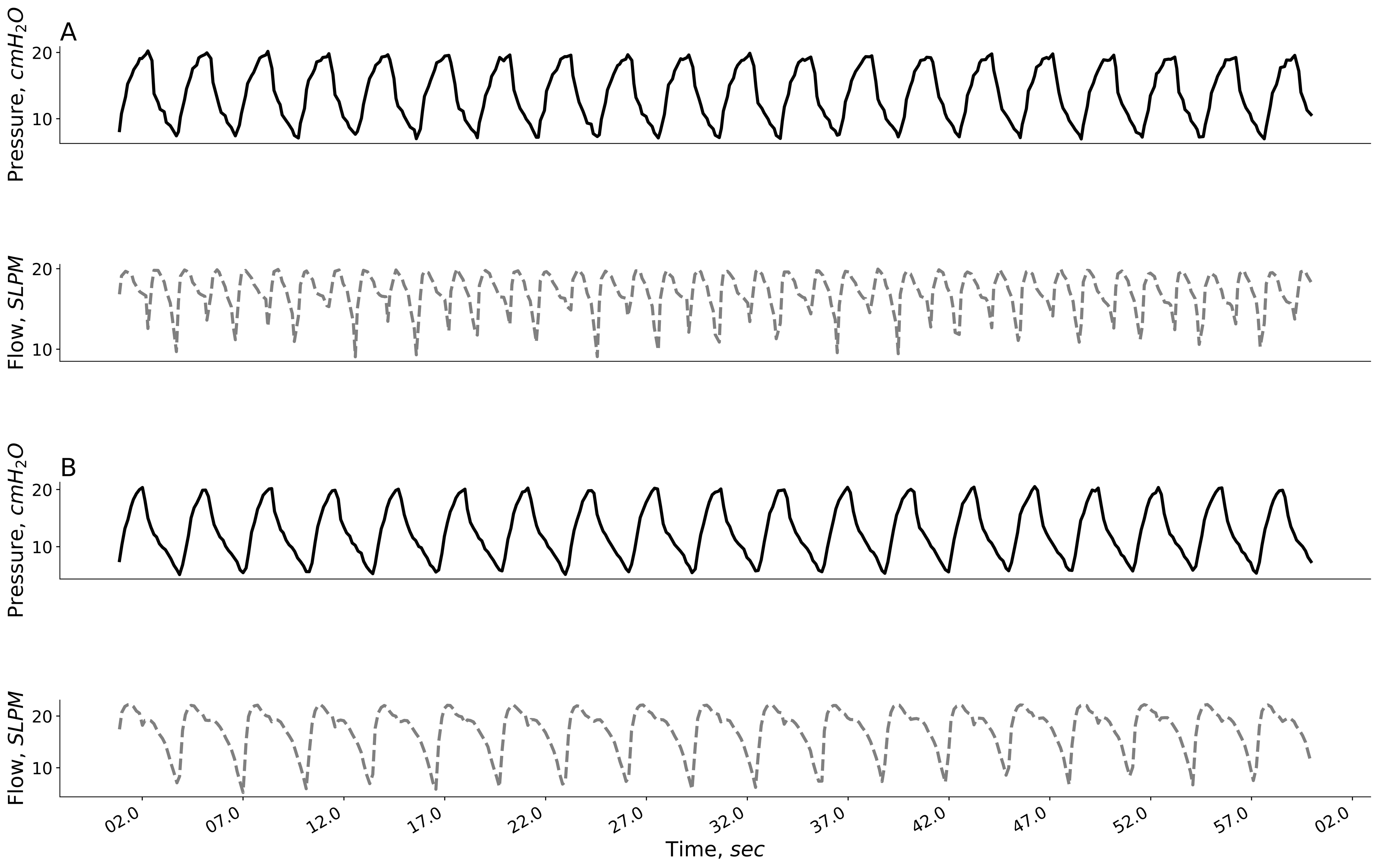

Supplement: S5 Fig — A: RapidVent prototype under test. B: Reference design. (TIF) [file pone.0244963.s006.tif]

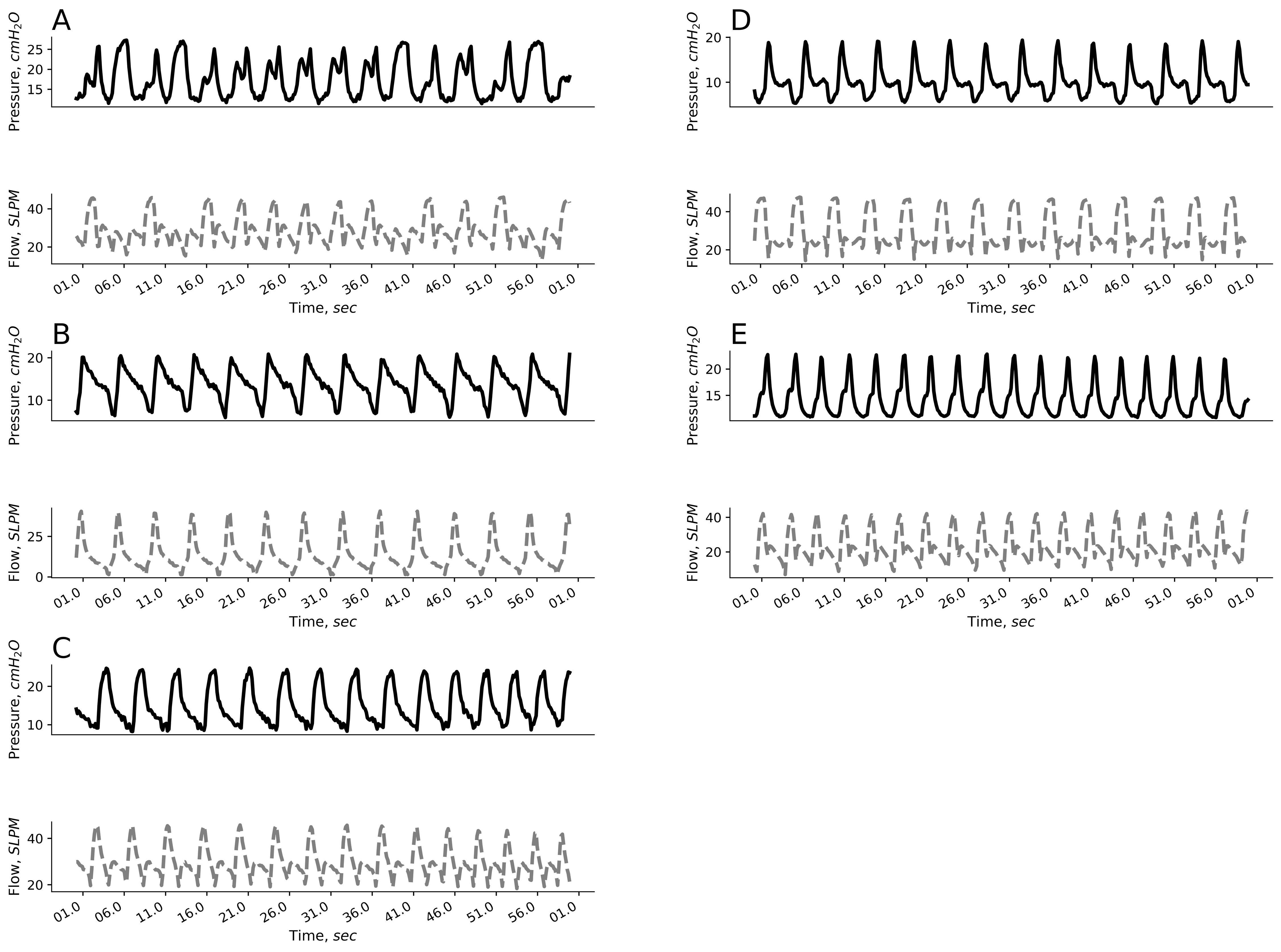

Supplement: S6 Fig — A: Animal 1 on RapidVent prototype 1 around mid-point. B: Animal 2 on RapidVent prototype 1 around mid-point. C: Animal 3 on RapidVent prototype I around mid-point. D: Animal 1 on RapidVent prototype II around mid-point. E: Animal 2 on RapidVent prototype 2 around mid-point. (TIF) [file pone.0244963.s007.tif]

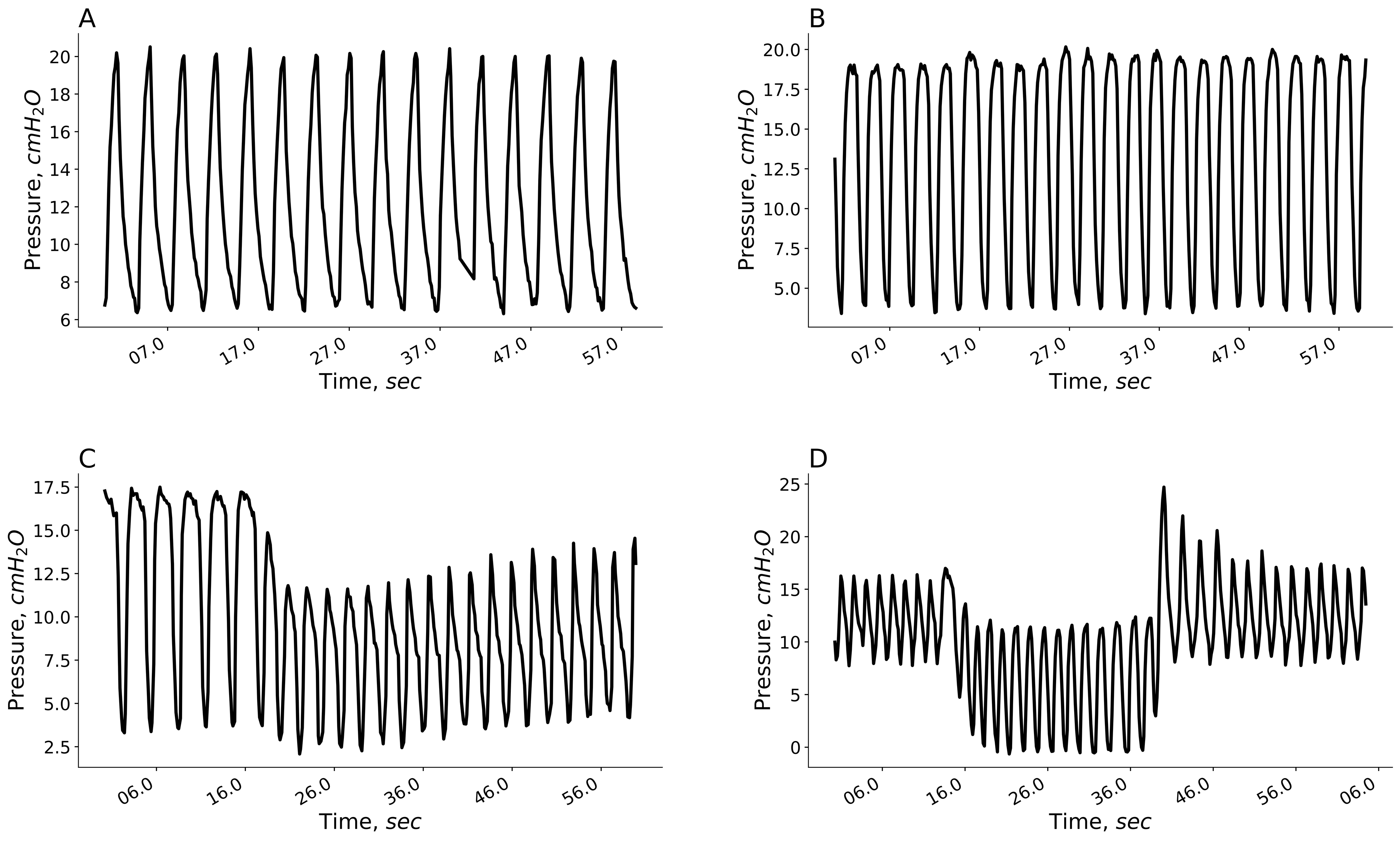

Supplement: S7 Fig — A: at test beginning. B: during the time where the animal was spontaneously breathing. C: where the rate dial was adjusted to fully open to allow more ventilation. Note the shape of the pressure curve restores to the shape observed in A, where the device was supporting the breathing. D: when 40 lbs. of sandbags were placed on the animal’s ribcage simulating restricted breathing. The pressure drops almost immediately in reaction to the available volume being restricted due to the weight. The ventilator was adjusted by increasing the PIP dial to compensate for the burden, and the settings stabilized in approximately 15 seconds after adjusting. (TIF) [file pone.0244963.s008.tif]

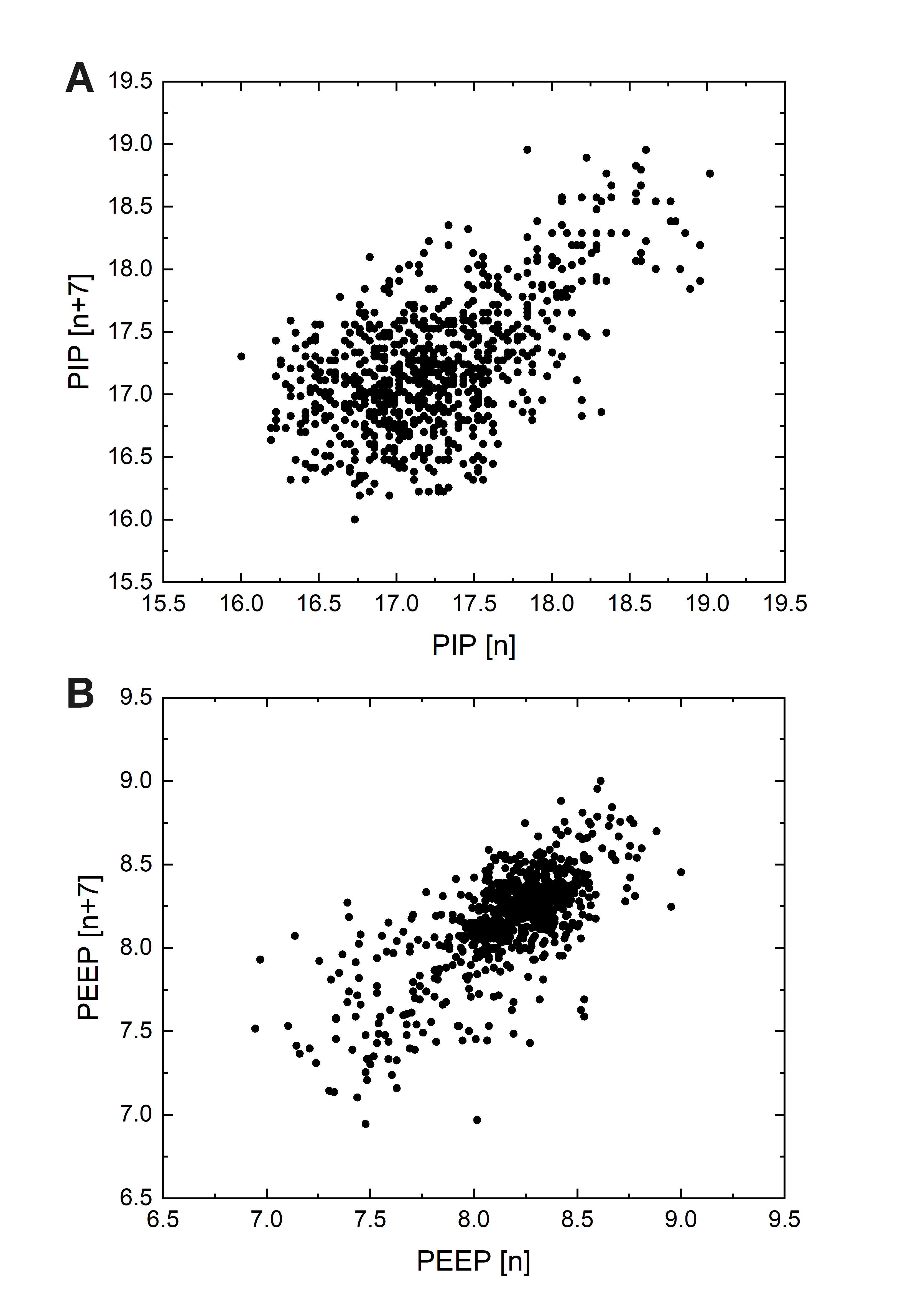

Supplement: S8 Fig — (A) Scatter plot of PIP at cycle [n] versus PIP at cycle [n+7] to observe the longitudinal variations of pressure during the testing. The PIP mean ± standard deviation is 17.25 ± 0.53 cm-H2O and the covariance [n, n+7] is 0.16. (B) Scatter plot of PEEP at cycle [n] versus PEEP at cycle [n+7] to observe the longitudinal variations of pressure during the testing. The PEEP mean ± standard deviation is 8.17 ± 0.3 cm-H2O and the covariance [n, n+7] is 0.06. (TIF) [file pone.0244963.s009.tif]
